# Post-abortion Complications: A Narrative Review for Emergency Clinicians

**DOI:** 10.5811/westjem.2022.8.57929

**Published:** 2022-10-23

**Authors:** Rachel E. Bridwell, Brit Long, Tim Montrief, Michael Gottlieb

**Affiliations:** * Madigan Army Medical Center, Department of Emergency Medicine, Tacoma, Washington; † Brooke Army Medical Center, Department of Emergency Medicine, Fort Sam Houston, Texas; ‡ Jackson Memorial Health System, Department of Emergency Medicine, Miami, Florida; § Rush University Medical Center, Department of Emergency Medicine, Chicago, Illinois

## Abstract

An abortion is a procedure defined by termination of pregnancy, most commonly performed in the first or second trimester. There are several means of classification, but the most important includes whether the abortion was maternally “safe” (performed in a safe, clean environment with experienced providers and no legal restrictions) or “unsafe” (performed with hazardous materials and techniques, by person without the needed skills, or in an environment where minimal medical standards are not met). Complication rates depend on the procedure type, gestational age, patient comorbidities, clinician experience, and most importantly, whether the abortion is safe or unsafe. Safe abortions have significantly lower complication rates compared to unsafe abortions. Complications include bleeding, retained products of conception, retained cervical dilator, uterine perforation, amniotic fluid embolism, misoprostol toxicity, and endometritis. Mortality rates for safe abortions are less than 0.2%, compared to unsafe abortion rates that range between 4.7–13.2%. History and physical examination are integral components in recognizing complications of safe and unsafe abortions, with management dependent upon the diagnosis. This narrative review provides a focused overview of post-abortion complications for emergency clinicians.

## INTRODUCTION

Abortion techniques and contraception have been described throughout history.^1^–^3^ In the current era, multiple countries place no restrictions on abortion, but most have an upper gestational age limit for when abortion can be performed, ranging from 6–24 weeks.^3^ However, as of 2021, 24 countries have issued a complete ban on abortions. The World Health Organization (WHO) classifies abortions as maternally “safe” or “unsafe”; “safe” abortion are ones performed in a setting where abortion laws are not restrictive, or if there is a formal law, safe abortion is still available.^3^,^4^ An “unsafe” abortion is performed by a person without the needed skills, performed with hazardous materials and techniques, or performed in an environment where minimal medical standards are not met.^3^,^5^–^7^

Unsafe abortions are a preventable pandemic, endangering females in locations where abortion is highly restricted by law or in countries where, even if legally permitted, safe abortion is not easily accessible.^8^,^9^ In this setting, females with an unintended pregnancy often self-induce abortions or obtain clandestine abortions from medical practitioners, paramedical workers, or traditional healers.^5^,^6^ Due to the risk of complications and potential risks associated with abortions, especially unsafe abortions, emergency clinicians must be able to recognize and manage these complications in the emergency department (ED) setting.

## METHODS

We searched PubMed and Google Scholar for articles using the keywords “abortion” OR “post-abortion” AND “complication” from January 1, 1950–June 7, 2022. We also searched the first 200 articles resulted by Google Scholar for each of the keywords. Articles reviewed included case reports and series, retrospective studies, prospective studies, systematic reviews and meta-analyses, and other narrative reviews. Literature searches were restricted to studies published in English. The gray literature including conference abstracts was not searched. Two emergency clinicians with experience in critical appraisal of the literature reviewed the articles and decided which to include for review by consensus, with a focus on emergency medicine-relevant articles. We preferentially selected systematic reviews and meta-analyses, followed by prospective studies, retrospective studies, case reports, and other narrative reviews. We included 123 resources for construction of this narrative review. Of these, there were zero guidelines, five systematic reviews and meta-analysis, 20 prospective studies, 27 retrospective studies, 26 case reports, and 35 narrative reviews. We also included 10 online resources from international organizations such as WHO and the US Centers for Disease Control and Prevention (CDC).

## DISCUSSION

### Epidemiology

Abortion rates vary based on several factors. From 2010–2014, the worldwide abortion rate was estimated at 35 per 1,000 females between the ages of 15–44 years.^10^,^11^ Rates approximate 37 per 1,000 in low- and middle-income countries (LMIC) and 27 per 1,000 in resource-rich countries.^10^,^11^ The highest rate of abortions occurs in those aged 20–29 years (18.5–19.1 per 1,000).^10^–^12^ In 2019, 629,898 abortions were reported to the CDC throughout the United States.^11^ Over 85% of these abortions occurred in unmarried patients, and abortion rates were highest in non-Hispanic Blacks.^10^–^13^ There are documented disparities in abortion rates, with higher rates in women of color, lower income, and less education, which may be associated with systemic hardships including reduced access to healthcare, racial discrimination, poorer living and working conditions, and greater stress.^13^

The majority of US abortions occur in the first trimester, with 92% performed at ≤13 weeks gestation, 8% at 14–20 weeks, and 1% at ≥ 21 weeks.^14^ Worldwide, second-trimester abortions comprise 10–15% of all abortions. Medication-induced abortion are responsible for 39% of abortions prior to nine weeks of gestation, while for those with gestational age ≥14 weeks, over 92% of abortions are surgical.^11^,^12^

Prior to 2022, over 26 million safe abortions and 20–25 million unsafe abortions were performed annually.^10^–^12^,^15^–^17^ Approximately 97% of unsafe abortion occur in LMIC.^9^ Complete data is limited due to the restrictions on abortions and the secrecy involved, but the highest rates appear to occur in Latin America and Africa at 31 per 1,000 females per year and 28 per 1,000 females per year, respectively.^18^ This is followed by Asia at rates of 11 per 1,000 females, although hospital admissions are highest in Asia, at 8.2 per 1,000 females.^18^

Patients can present to the ED after an abortion, some with complications from the abortion. Although abortion-related complications are rare in the US, there is a paucity of data regarding national-level estimates of abortion-related ED visits.^19^ Within California’s Medicaid program, 0.03% of abortions were followed by an immediate ambulance transfer to an ED, and 2.6% of abortions were followed by an abortion-related ED visit within six weeks of the abortion, while ED visit rates in New York and Philadelphia following an abortion were 0.3%, congruent with Planned Parenthood data from 2009.^20^–^22^

Complication rates depend on the procedure type, gestational age, patient comorbidities, clinician experience and, most importantly, whether the abortion was performed in a safe or unsafe manner.^8^,^9^,^19^,^23^ The majority of complications associated with abortion are minor, but major complications can occur including severe hemorrhage, endometritis, non-uterine organ injury, and disseminated intravascular coagulation (DIC). 8,9,19,23 A study evaluating 54,911 abortions found an overall complication rate of 2.1%.^19^ Medication abortions had a 5.2% complication rate (4.9% minor, 0.3% major), with rates of 1.3% in the first trimester and 1.5% for the second trimester.^19^ First-trimester aspiration had a complication rate of 2.3% (1.1% minor, 0.2% major).^19^ In the US, the overall mortality rate is less than 1 per 100,000 abortions performed, and in 2010 10 females died from a legally induced abortion.^11^,^12^,^21^,^24^,^25^ Mortality rates are lowest in the first nine weeks of gestation (<0.3 per 100,000 abortion), with an increase after this period (7 per 100,000 at 16–20 weeks of gestation and 11 per 100,000 at >21 weeks).^24^,^25^ This is similar to the rate of mortality associated with dental procedures (0–1.7 deaths per 100,000).^21^,^24^,^25^ Overall mortality rates for safe abortions are less than 0.2%, but for unsafe abortions the mortality rate is significant.^20^,^21^ Approximately 68,000 females die annually due to a complication from an unsafe abortion.^8^,^9^,^15^ Countries with less training of and access to abortion clinicians have higher maternal mortality rates.^25^ Annual maternal mortality rates associated with unsafe abortion range from 4.7–13.2%.^8^,^9^ In countries with significant resources, 30 females per 100,000 unsafe abortions die annually, but the incidence rises to 220 deaths per 100,000 unsafe abortions in settings with limited resources.^8^,^9^,^23^ Mortality associated with an unsafe abortion is most commonly due to septic abortion and hemorrhage.^8^,^9^,^23^

### Abortion Methods

There are several methods for safe abortions. The procedure may be medication-based or interventional, depending on the gestational age, patient preferences, experience of the clinician, and access to resources. Patients within the first trimester may undergo medical or interventional abortion (eg, aspiration). There are several differences between the two types, detailed in Table 1.^26^–^29^ In the second trimester, patients may undergo induction with medications or intervention with dilation and evacuation (D&E). Following termination of the pregnancy, patients typically experience vaginal bleeding similar to or slightly heavier than normal menstruation along with mild lower abdominal or pelvic cramping. Serum human chorionic gonadotropin (hCG) levels return to undetectable levels 7–60 days after the abortion.^26^

There are several methods by which unsafe abortions are performed.^3^,^6^ The method chosen depends on the patient, available resources, and any assistance the patient receives. The various forms are detailed in Table 2.

### Evaluation in the Emergency Department

The primary goal of the ED assessment is evaluation for dangerous post-abortion complications. A focused history and physical examination can provide important information and determine the need for further testing and treatment. History should include gestational history, estimated gestational age at the time of abortion, current symptoms (eg, bleeding, vaginal discharge, fever, chills, rigors, abdominal or pelvic pain), details of the abortion procedure (eg, procedure date, whether a surgical procedure was performed, medications used, whether any procedural complications occurred), and comorbidities.^6^ Medical history including known coagulopathy, diabetes, immunocompromised state, and prior abdominal and obstetric/gynecologic (OB/GYN) procedures should be obtained. Of note, females with a self-induced abortion may be hesitant to disclose the attempt due to perceived legal or social repercussions. Emergency clinicians must remain vigilant and inquire in a nonjudgmental fashion concerning any abortion attempt. Directed questions about where and how the abortion was performed are necessary to guide further evaluation and management.^6^ History should be obtained without the patient’s partner in the room.

Examination requires assessment of the patient’s hemodynamic status. Abdominal examination should assess for focal tenderness or evidence of peritonitis (eg, guarding or rigidity). Speculum and bimanual examinations should also be performed, evaluating for bleeding, vaginal discharge, trauma or laceration, uterine tone, tenderness, retained tissue, and masses.^6^ Laboratory analysis should include the following: complete blood count; electrolytes; renal and liver function; hCG level; coagulation panel, and blood type and screen (with crossmatch if bleeding) although this can be adjusted for the severity of presentation.^30^ If there is evidence of severe infection, blood and cervical cultures should be obtained, as well as lactic acid level.^31^,^32^ Fibrinogen, fibrin split products, and D-dimer should be obtained in patients with concern for DIC based on history and examination. The need for imaging evaluation is based on the suspected complication.^30^,^33^

Ultrasound can help identify retained products of conception (RPOC), ongoing pregnancy, ectopic masses, echogenic material within the uterus, hematoma formation, and intra-abdominal free fluid, which may suggest a uterine perforation, rupture, or vascular injury. An initial point-of-care ultrasound would be valuable to assess for intrauterine pregnancy and free fluid as part of the initial management, although many patients may need a more comprehensive radiologic ultrasound to assess for more advanced or complex findings. Computed tomography (CT) may help in the evaluation of uterine rupture, pelvic abscess, bowel injury, hematoma, or uterine myonecrosis.

### Bleeding

Vaginal bleeding is common after an abortion and is usually similar to or heavier than a normal menstrual cycle. Patients with medical abortions typically have more bleeding compared to surgical abortions and may present similarly to those having a spontaneous abortion.^6^,^29^ One study reported that blood loss ranged between 84–101 milliliters (mL) in patients undergoing safe medical abortion and 53 mL in abortion via aspiration.^34^ This bleeding does not typically require additional therapy or intervention, with less than 1% of first-trimester patients who underwent a safe abortion requiring acute intervention and 0.05% requiring a blood transfusion.^20^,^21^,^35^,^36^ Bleeding is usually bimodal, with moderate or heavy bleeding that is worse 3–8 hours after medication administration.^37^,^38^ After this time frame, bleeding lessens but can last up to 17 days, followed by bleeding 30–60 days that marks the resumption of menses.^39^–^42^ Initial bleeding most commonly lasts for 9–12 days following medical abortion, but this is less for those undergoing surgical abortion.^42^,^43^ In unsafe abortions, the rate of severe hemorrhage increases to 3%, with non-severe bleeding occurring in up to 44% of patients.^23^ The differential diagnosis for patients with post-abortion bleeding is demonstrated in Table 3.

Patients should seek medical evaluation if they soak through two pads per hour for two consecutive hours, which is suggestive of severe hemorrhage.^30^,^36^,^44^,^45^ Evaluation and management of hemorrhage in the post-abortion setting is similar to that of the postpartum period, with consideration of the differential listed in Table 3. If the physical examination does not reveal a readily apparent source (e.g., vaginal laceration), pelvic ultrasound should be performed to evaluate for RPOCs, uterine blood, or evidence of uterine perforation (eg, free fluid or air in the pelvis).^30^,^33^,^45^

Cervical or vaginal lacerations are typically repaired in the postoperative period. If a small laceration is discovered on ED evaluation, apply direct pressure or consider silver nitrate cauterization.^30^,^33^,^45^ Extensive lacerations may require OB/GYN consultation and placement of absorbable sutures.^30^,^33^,^45^ If an etiology is not discovered on physical examination or ultrasound, consider uterine atony. Uterine atony is treated with uterine massage and administration of uterotonic agents (Table 4). If uterotonic medications and bimanual massage are not effective, intrauterine tamponade can be performed, including a Bakri balloon or Foley catheter.^30^,^45^

Patients with severe bleeding and/or hemodynamic instability should receive blood products. Emergency consultation with OB/GYN and surgical specialists is recommended. Activation of a massive transfusion protocol may be required. Tranexamic acid (TXA) should also be considered.^30^ Literature has demonstrated that TXA may reduce the risk of postpartum hemorrhage and does not increase the risk of developing thromboses.^49^ If bleeding remains refractory to other therapies or in the setting of abnormal vascular bleeding, interventional radiology may need to perform uterine artery embolization.^30^,^50^–^52^ Some patients may also require surgical management in the operating room.^30^

### Retained products of conception

Retained products of conception occur more commonly with medical abortions compared to surgical abortions and are more common after the first trimester (2–10% of those undergoing abortion in the second trimester).^53^,^54^ Patients may report tissue passage even without passing the fetal tissue itself, as decidualized endometrium can shed with ongoing pregnancy.^6^,^29^ RPOCs are also more common in unsafe abortions, especially if self-induced, if the procedure is performed by an inexperienced individual or in later gestational ages, or uterine abnormality is present.^19^,^20^,^30^,^55^ Patients with RPOCs may present with vaginal bleeding, abdominal or pelvic pain, fever, and uterine tenderness.^55^ While bleeding is common after an abortion, large volume bleeding (≥2 pads per hour for ≥ 2 hours), sustained fever, worsening pain, or persistent pain lasting for multiple days is abnormal and should raise concern for RPOCs.^19^,^20^,^30^,^37^,^38^

**Figure 1:**
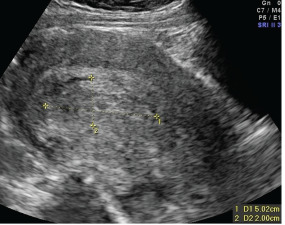
Ultrasound showing evidence of retained products of conception, demonstrated by echogenic, heterogeneous, and vascular intrauterine contents. Case courtesy of Dr Alexandra Stanislavsky, Radiopaedia.org, rID: 13852. Accessed at https://radiopaedia.org/cases/retained-products-of-conception-2?lang=us.

Ultrasound has a more limited ability to diagnose RPOCs as necrotic decidua and blood clots within the uterus following abortion can mimic RPOCs, with significant overlap between findings in asymptomatic and symptomatic patients.^56^,^57^ The uterus may demonstrate irregular and thickened lining with prominent color Doppler flow in patients with RPOCs, as well as those recovering after a successful abortion.^56^,^58^ However, a hyperechoic endometrial mass or solid component in the endometrium found on ultrasound in the setting of abnormal bleeding or evidence of infection is sensitive for RPOCs (Figure 1).^59^–^62^ Low-resistance Doppler flow within the myometrium or just below the endometrium is also suspicious for RPOCs.^62^,^63^ Emergent consultation with OB/GYN is recommended, as treatment includes RPOC removal through vacuum aspiration or D&E.^30^,^45^,^56^

### Misdiagnosed Ectopic Pregnancy

Ectopic pregnancy is associated with significant morbidity and mortality. However, ectopic pregnancy occurs in less than 1% of patients who present for abortion, which is close to three times lower than the overall rate of ectopic pregnancy.^64^–^66^ The literature suggests an ectopic pregnancy rate of 7=20 per 100,000 procedures.^21^,^67^ Ectopic pregnancy is most likely to occur in an abortion performed in a pregnancy of unknown location (ie, no yolk sac or fetal pole present on ultrasound).^68^ Patients with ectopic pregnancy most commonly present with abdominal pain or vaginal bleeding.^69^–^71^ Evaluation should include an ultrasound for intrauterine and extrauterine masses. In the post-abortion setting, ectopic pregnancy can be excluded by identifying products of conception after the aspiration.^68^ If an extrauterine mass is found on ultrasound, emergent consultation with OB/GYN is necessary.^69^–^71^

### Uterine Perforation

Uterine perforation is a potential complication of any intrauterine procedure and is the most common site of upper genital tract injury.^35^,^72^,^73^ Injury to the bowel, bladder, and surrounding vasculature may accompany uterine perforation.^35^,^72^–^75^ Data on these injuries is scarce, with three case series of 92 total uterine perforations reporting bowel or bladder injury in six cases.^76^–^78^ Overall, uterine perforation is uncommon, with rates ranging between 0.1–2.3% in safe medical abortions.^29^,^30^,^35^,^79^,^80^ Rates of uterine perforation are higher in unsafe abortions due to the instruments used and inexperience of the person performing the procedure.^23^ Factors associated with an increased risk of uterine perforation include surgeon inexperience and inadequate preoperative cervical dilation.^35^,^38^ Other factors include those that create difficulty in accessing the endometrial cavity (eg, cervical stenosis, uterine anteflexion/retroflexion) and those that alter the myometrial wall integrity and strength (eg, prior cesarean delivery, uterine scarring), particularly for those undergoing medication-induced abortion in the second trimester.^72^,^73^,^81^ While patients often experience mild-to-moderate abdominal or pelvic cramping for several hours after the procedure, continued and severe pain is atypical.^75^

**Figure 2:**
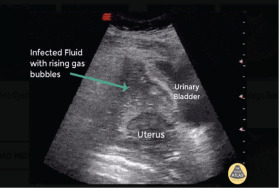
Ultrasound demonstrating pelvic free fluid and rising gas bubbles, indicative of uterine perforation. Image courtesy of Robert Jones, DO, POCUS Atlas. Available at https://www.thepocusatlas.com/ob-gyn-atlas.

Of note, patients can present up to several weeks after the procedure, which depends on the site of uterine injury and concomitant organ injuries.^82^,^83^ Symptoms include focal or diffuse abdominal/pelvic pain, abdominal distension, heavy or persistent vaginal bleeding, hematuria, and fever. Patients may also present with tachycardia and hypotension.^23^ Loops of bowel can become incarcerated within the uterine defect and result in bowel obstruction and subsequent perforation.^84^–^88^ Initial imaging includes ultrasound, which may demonstrate a defect in the uterine wall, abdominal free fluid, or abnormal contents within the uterus including fetal tissue (Figure 2).^86^,^89^

**Figure 3:**
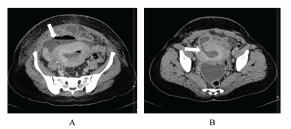
Computed tomography demonstrating a) fluid collection anterior to the uterus that communicates with the endometrial cavity and b) defect in the anterior wall of the uterus. Case courtesy of Dr Hidayatullah Hamidi, Radiopaedia.org, rID: 90743. Available at https://radiopaedia.org/cases/uterine-rupture-with-postpartum-infection?lang=us.

However, ultrasound should not be used to exclude uterine perforation; if the ultrasound is non-diagnostic, further imaging with CT of the abdomen/pelvis is recommended (Figure 3).^29^,^80^,^86^ Computed tomography is also recommended in the setting of suspected bowel perforation, as CT is highly sensitive and specific and can localize the site of perforation.^90^–^93^ If uterine perforation is suspected, emergent consultation with OB/GYN and general surgery is recommended. Patients with isolated uterine perforation can be managed surgically or expectantly, depending upon the patient’s hemodynamic status.^88^ Patients with concomitant bowel perforation require surgical specialist consultation, intravenous (IV) fluid resuscitation and symptomatic management, and broad-spectrum antibiotics.

### Retained Cervical Dilator

Osmotic dilators are typically placed the day prior to D&E. Complications are rare but can occur while the dilator is in place. These complications include bleeding, infection, rupture of membranes, and allergic reaction.^94^ Cases of retained cervical dilators have been reported, in which patients presented with pelvic pain and vaginal bleeding. In these cases, the retained dilator was found on imaging and removed surgically.^95^,^96^

### Amniotic Fluid Embolism

Amniotic fluid embolism (AFE) is a life-threatening obstetric complication following abortion or delivery.^97^,^98^ Patients develop sudden and refractory circulatory collapse with DIC, the latter of which occurs in up to 80% of patients.^97^,^99^ While AFE more commonly occurs in full-term deliveries, it can occur following abortion.^100^–^105^ An AFE secondary to abortion appears to be rare, but it accounted for approximately 5.5% of mortality in abortions within 2011–2013 Pregnancy Mortality Surveillance System analysis, with 1 of 111 AFEs occurring following an abortion.^106^,^107^

The American Society for Maternal-Fetal Medicine set forth four diagnostic criteria to improve recognition for this disease, which carries a mortality risk of 11–61%, The criteria include the following: 1) sudden hypotension or cardiopulmonary collapse; 2) DIC; 3) symptom development during labor and/or delivery of products of conception; and 4) absence of fever.^98^,^108^,^109^ Treatment requires prompt recognition of AFE, triggering immediate evacuation of the fetus or products of conception and aggressive maternal cardiopulmonary support to include fluid administration, vasopressor and inotropic support, as well as consideration of phosphodiesterase inhibition for right ventricular optimization.^110^ Venoarterial extracorporeal membrane oxygenation (ECMO) has demonstrated positive outcomes for severe cases in high-volume ECMO centers, although considerable risk is incurred with cannulation during profound coagulopathy.^111^,^112^ In a study of 10 AFEs treated with ECMO, there was a 70% survival-to-hospital-discharge rate.^113^

### Misoprostol Toxicity

Toxicity from misoprostol, a prostaglandin E analogue, is uncommon in safe abortion settings but is more likely in unsafe abortions. Toxic doses between 3–8 milligrams (mg) may result in severe fever, rigors, abdominal pain and cramping, vomiting and diarrhea, agitation, altered mental status, hypotension, hypoxemia, and rhabdomyolysis.^114^–^117^ These signs and symptoms typically develop quickly after initial ingestion, as misoprostol is completely absorbed from the stomach within 1.5 hours. Management includes removing remaining tablets from the stomach (e.g., gastric lavage) or vagina, along with supportive care including IV fluids and antiemetics. Vasopressors may be needed in those patients who are refractory to IV fluids. Symptoms typically resolve in 12 hours, but doses over 12 mg may result in multiorgan failure and death.^114^,^116^–^118^

### Post-abortion Infection and Endometritis

Septic abortion is defined by any uterine infection that complicates a spontaneous or induced abortion. This is a potential complication of both medication and surgical abortions and can be due to RPOCs or the procedure itself (eg, trauma, nonsterile technique). Importantly, post-abortion infection is a clinical diagnosis in the setting of a patient who presents with signs and symptoms of uterine infection following abortion. Septic abortion occurs in less than 0.4% of patients undergoing first trimester uterine aspiration and safe abortions, but this rate is much higher in those undergoing unsafe abortions.^20^,^21^ Non-severe infection occurs in 24% of those undergoing unsafe abortions, while severe infection occurs in 5.1%.^21^

The most common microbes include Enterobacteriaceae, streptococci, staphylococci, and enterococci, which are common endogenous vaginal and gastrointestinal flora.^31^ Other causative organisms include *Chlamydia trachomatis, Neisseria gonorrhoeae*, and *Trichomonas vaginalis* from pre-existing infections.^31^ Group A streptococcus and clostridium species can result in serious infections with rapid deterioration associated with toxic shock syndrome.^31^,^32^,^107^,^107^,^119^ Patients with post-abortion infections including endometritis typically present with pelvic and/or abdominal pain, nausea, vomiting, uterine tenderness, vaginal discharge and/or bleeding, and fever. Vaginal discharge is often sanguinopurulent.^20^,^31^,^32^ If history and examination suggest septic abortion, broad-spectrum antibiotics should be administered along with symptomatic management and resuscitation.

Antibiotic regimens are provided in Table 5.^20^,^31^,^32^ Consultation with OB/GYN is necessary if the diagnosis is suspected and should occur prior to imaging. Ultrasound can be used to evaluate for RPOCs, but a normal ultrasound should not be used to exclude septic abortion. Ultrasound may demonstrate intrauterine tissue, enhanced myometrial vascularity, hydrosalpinges, or an adnexal mass, which may suggest an abscess.^120^ Computed tomography may be used if the clinician is concerned for another condition or intra-abdominal abscess. If RPOCs are present, vacuum aspiration dilation and curettage is necessary.^32^ Patients may rapidly progress to acute respiratory distress syndrome, DIC, and acute renal injury.^8^,^9^,^19^,^23^,^121^,^122^

### Mental Health

While emergency clinicians must focus on the medical management of abortion complications, they must also be mindful of the patient’s mental health and wellbeing. Women who undergo an abortion may have increased rates of mental health issues as compared to women who do not; the highest risk population includes women undergoing an abortion who have pre-existing mental health issues.^123^ Regardless of the clinician’s personal views, they must approach these patients with compassion and address any mental health concerns.^123^

## LIMITATIONS

This is a narrative review, and thus no pooling of data from individual studies was conducted. Neither did we assess article quality or risk of bias. Much of the included literature consists of studies conducted in non-emergent settings; thus, generalizing these studies to the ED setting is challenging. Much of the information and resources come from society guidelines.

## CONCLUSION

Abortion complications present a spectrum of emergencies ranging from small lacerations to life-threatening complications requiring immediate control. Unsafe abortions have a far higher rate of complications. Complications include bleeding, retained products of conception, retained cervical dilator, uterine perforation, amniotic fluid embolism, misoprostol toxicity, and endometritis. Supportive and nonjudgmental history and physical examination are integral in recognizing complications of safe abortions as well as issues that arise from unsafe abortions. Prompt recognition of the specific emergency with immediate stabilization and potential specialist consultation can mitigate morbidity and mortality.

## Supplementary Information



## Figures and Tables

**Table 1:** Safe abortion types.

1st trimester: Medication (Induction)	- Regimens: Mifepristone 200 mg plus misoprostol 800 mcg or misoprostol only - Typically used up to 11 weeks gestational age - Vaginal bleeding begins 1–4 hours after medication administration, with pregnancy expulsion occurs 3–8 hours after medication administration - Side effects can include abdominal cramping, vaginal bleeding, brief low-grade fever, headache, dizziness, nausea, vomiting, and diarrhea - Efficacy approximates 95–98% - Higher complication rate due to risk of failure and retained tissue
1st trimester: Uterine aspiration	- Procedure includes dilation of the cervix, insertion of a cannula into the uterine cavity, and aspiration of uterine contents - Cervical ripening agent (e.g., misoprostol) can be used - Used up to 14 weeks gestation - Efficacy approximates 99% - Typically requires local anesthesia and/or sedation
2nd trimester: Medication (Induction)	- Regimens: misoprostol (most common), mifepristone, misoprostol and mifepristone, oxytocin, carboprost, sulprostone - Allows for expulsion of intact fetus - Higher risk of complications compared to interventional measures, including hemorrhage and retained products - Approximately 8–10% of cases require intervention for further removal - May require 24 hours or longer before pregnancy expulsion is completed
2nd trimester: Dilation and evacuation	- Account for the majority of second-trimester abortions - Short procedure time (<30 minutes once cervix is dilated) - Higher efficacy rates compared to second-trimester medical abortions - Risk of uterine perforation - Prophylactic antibiotics are administered

*mg*, milligram; *mcg*, microgram.

**Table 2:** Unsafe abortion types and complications.

Forms of unsafe abortion	Abortion Complications*
-Oral or injectable material: chloroquine, detergents, hormones, kerosene, lead, metal salts, oral contraceptive pills, phosphorus, teas/herbal remedies, turpentine, uterine stimulants (misoprostol or oxytocin) -Preparation placed in the cervix, vagina, or rectum: enemas, herbal preparations, misoprostol, potassium permanganate tablets -Intrauterine instrumentation: catheter insertion and infusion of substance (alcohol, saline), foreign body insertion (knitting needles, stitch hook, coat hanger, air blown through a syringe) -Transcervical introduction of substances: Cresol, phenol, soap -Trauma to abdomen or back: Abdominal massage, jumping from a height, lifting heavy weights, self-inflicted blows	-Bleeding -Retained products of conception -Misdiagnosed ectopic pregnancy -Uterine perforation -Retained cervical dilator -Amniotic fluid embolism -Misoprostol/substance toxicity -Post-abortion infection and endometritis

* These complications may occur in both safe and unsafe abortions.

**Table 3:** Post-abortion bleeding etiologies.

More common: -Cervical or vaginal canal laceration -Coagulopathy -Retained products of conception -Uterine atony -Uterine perforation Less common: -Abnormal placenta location -Undiagnosed ectopic or heterotopic pregnancy -Uterine arteriovenous malformation -Uterine artery pseudoaneurysm

**Table 4:** Management of uterine atony.

Intervention	Dosing, route, and side effects
Initial^30^	Bimanual massage ^*^Ensure that the placenta is evacuated completely
Medical	Misoprostol 600 mcg SL or 1000 mcg PR^46^ Methylergonovine 0.2 mg IM^47^ *Avoid in patients with hypertension Carboprost 250 mcg IM^48^ *Avoid in patients with asthma as well as cardiac, hepatic, pulmonary, and renal disease
Physical tamponade^30^,^45^	Bakri balloon–fill with 500 mL of warm normal saline Foley balloon–fill with 50–60 mL

*mcg*, microgram; *SL*, sublingual; *PR*, by rectum; *IM*, intramuscular; *ml*, milliliter.

**Table 5:** Antibiotic regimens for post-abortion infection or endometritis.

Imipenem 500 mg IV
Piperacillin-tazobactam 4.5 g IV
Levofloxacin 500 mg IV + metronidazole 500 mg IV
Gentamicin 5 mg/kg IV + ampicillin 2 g IV + metronidazole 500 mg IV
Gentamicin 5 mg/kg IV + ampicillin 2g IV + clindamycin 900 mg IV

*IV*, intravenous; *mg*, milligram; *kg*, kilogram; *g*, gram.
