# Supplementary material for: Post-abortion Complications: A Narrative Review for Emergency Clinicians
Source: West J Emerg Med. 2022 Oct 23;23(6):919–25. doi: 10.5811/westjem.2022.8.57929 (PMC9683756; doi:10.5811/westjem.2022.8.57929)
Supplement: Supplementary file 1 [file wjem-23-919-s001.docx]

**REFERENCES**

1. Joffe C. Abortion and Medicine: A Sociopolitical History. In: *Management of Unintended and Abnormal Pregnancy: Comprehensive Abortion Care*. John Wiley and Sons; 2009:1-9. doi:10.1002/9781444313031.ch1
2. Suojanen JN. Riddle, John M. Contraception and Abortion from the Ancient World to the Renaissance. *Natl Cathol Bioeth Q*. 2001;1(1):116-118. doi:10.5840/ncbq20011187
3. Abortion. https://www.who.int/news-room/fact-sheets/detail/abortion. Accessed June 27, 2022.
4. World Population Review. Countries Where Abortion Is Illegal 2022. World Population Review. https://worldpopulationreview.com/country-rankings/countries-where-abortion-is-illegal. Published 2022. Accessed June 27, 2022.
5. Haddad LB, Nour NM. Unsafe Abortion: Unnecessary Maternal Mortality. *Rev Obstet Gynecol*. 2009;2(2):122. /pmc/articles/PMC2709326/. Accessed June 27, 2022.
6. Harris LH, Grossman D. Complications of Unsafe and Self-Managed Abortion. *N Engl J Med*. 2020;382(11):1029-1040. doi:10.1056/NEJMRA1908412
7. Fawcus SR. Maternal mortality and unsafe abortion. *Best Pract Res Clin Obstet Gynaecol*. 2008;22(3):533-548. doi:10.1016/j.bpobgyn.2007.10.006
8. Say L, Chou D, Gemmill A, et al. Global causes of maternal death: a WHO systematic analysis. *Lancet Glob Heal*. 2014;2(6). doi:10.1016/S2214-109X(14)70227-X
9. Ganatra B, Gerdts C, Rossier C, et al. Global, regional, and subregional classification of abortions by safety, 2010–14: estimates from a Bayesian hierarchical model. *Lancet*. 2017;390(10110):2372-2381. doi:10.1016/S0140-6736(17)31794-4/ATTACHMENT/D5100FC1-A915-4107-87DC-9DE7C3ADBFB3/MMC1.PDF
10. Jones RK, Witwer E, Jerman J. Abortion Incidence and Service Availability in the United States, 2017. September 2019. doi:10.1363/2019.30760
11. Kortsmit K, Jatlaoui TC, Mandel MG, et al. Abortion Surveillance — United States, 2018. *MMWR Surveill Summ*. 2021;69(7):1-30. doi:10.15585/MMWR.SS6907A1
12. Kortsmit K, Mandel MG, Reeves JA, et al. Abortion Surveillance — United States, 2019. *MMWR Surveill Summ*. 2021;70(9):1-29. doi:10.15585/MMWR.SS7009A1
13. Dehlendorf C, Harris LH, Weitz TA. Disparities in abortion rates: a public health approach. *Am J Public Health*. 2013;103(10):1772-1779. doi:10.2105/AJPH.2013.301339
14. Jatlaoui TC, Boutot ME, Mandel MG, et al. Abortion Surveillance — United States, 2015. *MMWR Surveill Summ*. 2020;67(13):1-45. doi:10.15585/MMWR.SS6713A1
15. Sedgh G, Bearak J, Singh S, et al. Abortion incidence between 1990 and 2014: global, regional, and subregional levels and trends. *Lancet*. 2016;388(10041):258-267. doi:10.1016/S0140-6736(16)30380-4
16. Prata N, Sreenivas A, Vahidnia F, Potts M. Saving maternal lives in resource-poor settings: Facing reality. *Health Policy (New York)*. 2009;89(2):131-148. doi:10.1016/j.healthpol.2008.05.007
17. Addante AN, Eisenberg DL, Valentine MC, Leonard J, Maddox KEJ, Hoofnagle MH. The association between state-level abortion restrictions and maternal mortality in the United States, 1995-2017. *Contraception*. 2021;104(5):496-501. doi:10.1016/j.contraception.2021.03.018
18. World Health Organization. WHO | Unsafe abortion: global and regional estimates of the incidence of unsafe abortion and associated mortality in 2008. *Who*. 2014;5th editio:1-67. http://whqlibdoc.who.int/publications/2011/9789241501118_eng.pdf. Accessed June 27, 2022.
19. Upadhyay UD, Johns NE, Barron R, et al. Abortion-related emergency department visits in the United States: An analysis of a national emergency department sample. *BMC Med*. 2018;16(1). doi:10.1186/s12916-018-1072-0
20. Upadhyay UD, Desai S, Zlidar V, et al. Incidence of emergency department visits and complications after abortion. *Obstet Gynecol*. 2015;125(1):175-183. doi:10.1097/AOG.0000000000000603
21. Cleland K, Creinin MD, Nucatola D, Nshom M, Trussell J. Significant adverse events and outcomes after medical abortion. *Obstet Gynecol*. 2013;121(1):166-171. doi:10.1097/AOG.0b013e3182755763
22. Bennett IM, Baylson M, Kalkstein K, Gillespie G, Bellamy SL, Fleischman J. Early abortion in family medicine: Clinical outcomes. *Ann Fam Med*. 2009;7(6):527-533. doi:10.1370/afm.1051
23. Adler AJ, Filippi V, Thomas SL, Ronsmans C. Quantifying the global burden of morbidity due to unsafe abortion: Magnitude in hospital-based studies and methodological issues. In: *International Journal of Gynecology and Obstetrics*. Vol 118. Int J Gynaecol Obstet; 2012. doi:10.1016/S0020-7292(12)60003-4
24. Bartlett LA, Berg CJ, Shulman HB, et al. Risk factors for legal induced abortion-related mortality in the United States. *Obstet Gynecol*. 2004;103(4):729-737. doi:10.1097/01.AOG.0000116260.81570.60
25. Raymond EG, Grossman D, Weaver MA, Toti S, Winikoff B. Mortality of induced abortion, other outpatient surgical procedures and common activities in the United States. *Contraception*. 2014;90(5):476-479. doi:10.1016/j.contraception.2014.07.012
26. World Health Organization. *Abortion Care Guideline*.; 2022. https://apps.who.int/iris/handle/10665/349316. Accessed June 27, 2022.
27. National Abortion Federation. 2020 Clinical Policy Guidelines for Abortion Care. 2020:1-68. www.prochoice.org. Accessed June 27, 2022.
28. Robson SC, Kelly T, Howel D, et al. Randomised preference trial of medical versus surgical termination of pregnancy less than 14 weeks’ gestation (TOPS). *Health Technol Assess (Rockv)*. 2009;13(53):1-124. doi:10.3310/hta13530
29. Grossman D, Grindlay K, Buchacker T, Lane K, Blanchard K. Effectiveness and acceptability of medical abortion provided through telemedicine. *Obstet Gynecol*. 2011;118(2):296-303. doi:10.1097/AOG.0b013e318224d110
30. Kerns J, Steinauer J. Management of postabortion hemorrhage. In: *Contraception*. Vol 87. Contraception; 2013:331-342. doi:10.1016/j.contraception.2012.10.024
31. Rahangdale L. Infectious complications of pregnancy termination. *Clin Obstet Gynecol*. 2009;52(2):198-204. doi:10.1097/GRF.0b013e3181a2b6dd
32. Eschenbach DA. Treating spontaneous and induced septic abortions. *Obstet Gynecol*. 2015;125(5):1042-1048. doi:10.1097/AOG.0000000000000795
33. Yonke N, Leeman LM. First-trimester surgical abortion technique. *Obstet Gynecol Clin North Am*. 2013;40(4):647-670. doi:10.1016/j.ogc.2013.08.006
34. Chan YF, Ho PC, Ma HK. Blood loss in termination of early pregnancy by vacuum aspiration and by combination of mifepristone and gemeprost. *Contraception*. 1993;47(1):85-95. doi:10.1016/0010-7824(93)90111-J
35. White K, Carroll E, Grossman D. Complications from first-trimester aspiration abortion: a systematic review of the literature. *Contraception*. 2015;92(5):422-438. doi:10.1016/j.contraception.2015.07.013
36. Creinin MD, Grossman DA. Medical management of first-trimester abortion. *Contraception*. 2014;89(3):148-161. doi:10.1016/j.contraception.2014.01.016
37. Bryant AG, Regan E, Stuart G. An overview of medical abortion for clinical practice. *Obstet Gynecol Surv*. 2014;69(1):39-45. doi:10.1097/OGX.0000000000000017
38. Wu YM, Gomez-Alzugaray M, Haukkamaa M, et al. Comparison of two doses of mifepristone in combination with misoprostol for early medical abortion: A randomised trial. *BJOG An Int J Obstet Gynaecol*. 2000;107(4):524-530. doi:10.1111/j.1471-0528.2000.tb13273.x
39. Christin-Maitre S, Bouchard P, Spitz IM. Medical termination of pregnancy. Wood AJJ, ed. *N Engl J Med*. 2000;342(13):946-956. doi:10.1056/NEJM200003303421307
40. Hertzen H, Honkanen H, Piaggio G, et al. WHO multinational study of three misoprostol regimens after mifepristone for early medical abortion. I: Efficacy. *BJOG An Int J Obstet Gynaecol*. 2003;110(9):808-818. doi:10.1111/j.1471-0528.2003.02430.x
41. Honkanen H, Piaggio G, Von Hertzen H, et al. WHO multinational study of three misoprostol regimens after mifepristone for early medical abortion. II: Side effects and women’s perceptions. *BJOG An Int J Obstet Gynaecol*. 2004;111(7):715-725. doi:10.1111/j.1471-0528.2004.00153.x
42. Davis AR, Hendlish SK, Westhoff C, et al. Bleeding patterns after misoprostol vs surgical treatment of early pregnancy failure: results from a randomized trial. *Am J Obstet Gynecol*. 2007;196(1):31.e1-31.e7. doi:10.1016/j.ajog.2006.07.053
43. Trinder J, Brocklehurst P, Porter R, Read M, Vyas S, Smith L. Management of miscarriage: Expectant, medical, or surgical? Results of randomised controlled trial (miscarriage treatment (MIST) trial). *Br Med J*. 2006;332(7552):1235-1238. doi:10.1136/bmj.38828.593125.55
44. Kruse B, Poppema S, Creinin MD, Paul M. Management of side effects and complications in medical abortion. *Am J Obstet Gynecol*. 2000;183(2 Suppl):S65-S75. doi:10.1067/MOB.2000.107946
45. Orlowski MH, Soares WE, Kerrigan KL, Zerden ML. Management of Postabortion Complications for the Emergency Medicine Clinician. *Ann Emerg Med*. 2021;77(2):221-232. doi:10.1016/j.annemergmed.2020.09.008
46. Co. GDS and. Cytotec® (misoprostol) Package Insert.
47. Novartis. Methergine ® (methylergonovine maleate) Package insert.
48. Pfizer. Hemabate ® carboprost tromethamine injection, package insert.
49. WOMAN Trial Collaborators H, Roberts I, Fawole B, et al. Effect of early tranexamic acid administration on mortality, hysterectomy, and other morbidities in women with post-partum haemorrhage (WOMAN): an international, randomised, double-blind, placebo-controlled trial. *Lancet (London, England)*. 2017;389(10084):2105-2116. doi:10.1016/S0140-6736(17)30638-4
50. Vegas G, Illescas T, Munoz M. Selective pelvic arterial embolizationin the managemetn of obstetric hemorrhage. *Eur J Obstet Gynecol Reprod Bio*. 2006 Jul;127(1):68-72. doi:10.1016/j.ejogrb.2005.09.008
51. Steinauer JE, Diedrich JT, Wilson MW, Darney PD, Vargas JE, Drey EA. Uterine artery embolization in postabortion hemorrhage. *Obstet Gynecol*. 2008;111(4):881-889. doi:10.1097/AOG.0B013E3181685780
52. Gonsalves M, Belli A. The role of interventional radiology in obstetric hemorrhage. *Cardiovasc Intervent Radiol*. 2010;33(5):887-895. doi:10.1007/s00270-010-9864-4
53. Rooks J, Cates Jr W. Abortion methods: morbidity, costs and emotional impact. 3. Emotional impact of D&E vs. instillation - PubMed. *Fam Plann Perspect*. 1977;9(6):277-277. https://pubmed.ncbi.nlm.nih.gov/923758/. Accessed June 27, 2022.
54. Cui R, Li M, Lu J, Bai H, Zhang Z. Management strategies for patients with placenta accreta spectrum disorders who underwent pregnancy termination in the second trimester: a retrospective study. *BMC Pregnancy Childbirth*. 2018;18(1). doi:10.1186/S12884-018-1935-6
55. Wolman I, Altman E, Faith G, et al. Combined clinical and ultrasonographic work-up for the diagnosis of retained products of conception. *Fertil Steril*. 2009;92(3):1162-1164. doi:10.1016/J.FERTNSTERT.2009.01.087
56. Dayananda I, Maurer R, Fortin J, Goldberg AB. Medical abortion follow-up with serum human chorionic gonadotropin compared with ultrasonography: a randomized controlled trial. *Obstet Gynecol*. 2013;121(3):607-613. doi:10.1097/AOG.0B013E3182839FDA
57. Cowett AA, Cohen LS, Lichtenberg ES, Stika CS. Ultrasound evaluation of the endometrium after medical termination of pregnancy. *Obstet Gynecol*. 2004;103(5 Pt 1):871-875. doi:10.1097/01.AOG.0000124782.69622.48
58. McEwing RL, Anderson NG, Meates JBA, Allen RB, Phillipson GTM, Wells JE. Sonographic appearances of the endometrium after termination of pregnancy in asymptomatic versus symptomatic women. *J Ultrasound Med*. 2009;28(5):579-586. doi:10.7863/JUM.2009.28.5.579
59. Ben-Ami I, Schneider D, Maymon R, Vaknin Z, Herman A, Halperin R. Sonographic versus clinical evaluation as predictors of residual trophoblastic tissue. *Hum Reprod*. 2005;20(4):1107-1111. doi:10.1093/HUMREP/DEH689
60. Abbasi S, Jamal A, Eslamian L, Marsousi V. Role of clinical and ultrasound findings in the diagnosis of retained products of conception. *Ultrasound Obstet Gynecol*. 2008;32(5):704-707. doi:10.1002/uog.5391
61. Sawyer E, Ofuasia E, Ofili-Yebovi D, Helmy S, Gonzalez J, Jurkovic D. The value of measuring endometrial thickness and volume on transvaginal ultrasound scan for the diagnosis of incomplete miscarriage. *Ultrasound Obstet Gynecol*. 2007;29(2):205-209. doi:10.1002/UOG.3914
62. Van Den Bosch T, Daemen A, Van Schoubroeck D, Pochet N, De Moor B, Timmerman D. Occurrence and outcome of residual trophoblastic tissue: a prospective study. *J Ultrasound Med*. 2008;27(3):357-361. doi:10.7863/JUM.2008.27.3.357
63. Alcázar JL. Transvaginal ultrasonography combined with color velocity imaging and pulsed Doppler to detect residual trophoblastic tissue. *Ultrasound Obstet Gynecol*. 1998;11(1):54-58. doi:10.1046/J.1469-0705.1998.11010054.X
64. Center for Disease Control. Ectopic pregnancy--United States, 1988-1989. *MMWR Morb Mortal Wkly Rep*. 1992;41(32):591-594. https://pubmed.ncbi.nlm.nih.gov/1640927/. Accessed June 27, 2022.
65. Ulmann A, Silvestre L, Chemama L, et al. Medical termination of early pregnancy with mifepristone (RU 486) followed by a prostaglandin analogue. Study in 16,369 women. *Acta Obstet Gynecol Scand*. 1992;71(4):278-283. doi:10.3109/00016349209021052
66. Edwards J, Carson SA. New technologies permit safe abortion at less than six weeks’ gestation and provide timely detection of ectopic gestation. *Am J Obstet Gynecol*. 1997;176(5):1101-1106. doi:10.1016/S0002-9378(97)70410-1
67. Shannon C, Brothers LP, Philip NM, Winikoff B. Ectopic pregnancy and medical abortion. *Obstet Gynecol*. 2004;104(1):161-167. doi:10.1097/01.AOG.0000130839.61098.12
68. Pontius E, Vieth JT. Complications in Early Pregnancy. *Emerg Med Clin North Am*. 2019;37(2):219-237. doi:10.1016/J.EMC.2019.01.004
69. Taran FA, Kagan KO, Hübner M, Hoopmann M, Wallwiener D, Brucker S. The Diagnosis and Treatment of Ectopic Pregnancy. *Dtsch Arztebl Int*. 2015;112(41):693-704. doi:10.3238/ARZTEBL.2015.0693
70. Hendriks E, Rosenberg R, Prine L. Ectopic Pregnancy: Diagnosis and Management. *Am Fam Physician*. 2020;101(10):599-606. https://pubmed.ncbi.nlm.nih.gov/32412215/. Accessed June 27, 2022.
71. Robertson JJ, Long B, Koyfman A. Emergency Medicine Myths: Ectopic Pregnancy Evaluation, Risk Factors, and Presentation. *J Emerg Med*. 2017;53(6):819-828. doi:10.1016/J.JEMERMED.2017.08.074
72. Gentile GP, Siegler AM. Inadvertent intestinal biopsy during laparoscopy and hysteroscopy: a report of two cases. *Fertil Steril*. 1981;36(3):402-404. doi:10.1016/S0015-0282(16)45746-8
73. Sullivan B, Kenney P, Seibel M. Hysteroscopic resection of fibroid with thermal injury to sigmoid. *Obs Gynecol*. 1992;80(3 pt 2):546-547. https://pubmed.ncbi.nlm.nih.gov/1495733/. Accessed June 27, 2022.
74. Hefler L, Lemach A, Seebacher V, Polterauer S, Tempfer C, Reinthaller A. The intraoperative complication rate of nonobstetric dilation and curettage. *Obstet Gynecol*. 2009;113(6):1268-1271. doi:10.1097/AOG.0B013E3181A66F91
75. Darney PD, Sweet RL. Routine intraoperative ultrasonography for second trimester abortion reduces incidence of uterine perforation. *J Ultrasound Med*. 1989;8(2):71-75. doi:10.7863/jum.1989.8.2.71
76. Aydeniz B, Gruber I V., Schauf B, Kurek R, Meyer A, Wallwiener D. A multicenter survey of complications associated with 21,676 operative hysteroscopies. *Eur J Obstet Gynecol Reprod Biol*. 2002;104(2):160-164. doi:10.1016/S0301-2115(02)00106-9
77. Agostini A, Cravello L, Bretelle F, Shojai R, Roger V, Blanc B. Risk of uterine perforation during hysteroscopic surgery. *J Am Assoc Gynecol Laparosc*. 2002;9(3):264-267. doi:10.1016/S1074-3804(05)60401-X
78. Jansen FW, Vredevoogd CB, Van Ulzen K, Hermans J, Trimbos JB, Trimbos-Kemper TCM. Complications of hysteroscopy: a prospective, multicenter study. *Obstet Gynecol*. 2000;96(2):266-270. doi:10.1016/S0029-7844(00)00865-6
79. Kaali SG, Szigetvari IA, Bartfai GS. The frequency and management of uterine perforations during first-trimester abortions. *Am J Obstet Gynecol*. 1989;161(2):406-408. doi:10.1016/0002-9378(89)90532-2
80. Chen L, Lai S, Lee W, Leon N. Uterine perforation during elective first trimester abortions: a 13-year review. *Singapore Med J*. 1995;36(1):63-67. https://pubmed.ncbi.nlm.nih.gov/7570139/. Accessed June 27, 2022.
81. Goyal V. Uterine rupture in second-trimester misoprostol-induced abortion after cesarean delivery: a systematic review. *Obstet Gynecol*. 2009;113(5):1117-1123. doi:10.1097/AOG.0B013E31819DBFE2
82. Bhole S, Harris ME, Sistrom CL, Shifrin RY, Mulvihill MS, Moawad NS. Retained products of conception through a perforated uterine wall following elective abortion: a unique case report. *Emerg Radiol*. 2012;19(5):477-481. doi:10.1007/S10140-012-1040-5
83. Chang HM, Shen CJ, Lin CY, Tsai EM. Uterine perforation and bowel incarceration following surgical abortion during the first trimester. *Taiwan J Obstet Gynecol*. 2008;47(4):448-450. doi:10.1016/S1028-4559(09)60016-4
84. Istre O. Managing bleeding, fluid absorption and uterine perforation at hysteroscopy. *Best Pract Res Clin Obstet Gynaecol*. 2009;23(5):619-629. doi:10.1016/J.BPOBGYN.2009.03.003
85. Tong S, Jeflfares J, Lopes R, Vollenhoven B. Delayed presentation of uterine perforation and haemorrhagic shock 10 days after surgical termination of pregnancy. *Aust N Z J Obstet Gynaecol*. 2001;41(3):335-336. doi:10.1111/J.1479-828X.2001.TB01241.X
86. Shulman SG, Bell CL, Hampf FE. Uterine perforation and small bowel incarceration: sonographic and surgical findings. *Emerg Radiol*. 2006;13(1):43-45. doi:10.1007/S10140-006-0499-3
87. Dignac A, Novellas S, Fournol M, Caramella T, Bafghi A, Chevallier P. Incarceration of the appendix complicating a uterine perforation following surgical abortion: CT aspects. *Emerg Radiol*. 2008;15(4):267-269. doi:10.1007/S10140-007-0679-9
88. Coughlin LM, Sparks DA, Chase DM, Smith J. Incarcerated small bowel associated with elective abortion uterine perforation. *J Emerg Med*. 2013;44(3). doi:10.1016/J.JEMERMED.2012.02.071
89. Derr C, Henry M. Bedside ultrasound in the diagnosis of uterine rupture following surgical abortion. *Emerg Radiol*. 2013;20(2):165-167. doi:10.1007/S10140-012-1069-5
90. Del Gaizo AJ, Lall C, Allen BC, Leyendecker JR. From esophagus to rectum: a comprehensive review of alimentary tract perforations at computed tomography. *Abdom Imaging*. 2014;39(4):802-823. doi:10.1007/S00261-014-0110-4
91. Singh JP, Steward MJ, Booth TC, Mukhtar H, Murray D. Evolution of imaging for abdominal perforation. *Ann R Coll Surg Engl*. 2010;92(3):182. doi:10.1308/003588410X12664192075251
92. Pouli S, Kozana A, Papakitsou I, Daskalogiannaki M, Raissaki M. Gastrointestinal perforation: clinical and MDCT clues for identification of aetiology. *Insights Imaging*. 2020;11(1). doi:10.1186/S13244-019-0823-6
93. West AB. The pathology of diverticulitis. *J Clin Gastroenterol*. 2008;42(10):1137-1138. doi:10.1097/MCG.0b013e3181862a9f
94. Lichtenberg ES. Complications of osmotic dilators. *Obstet Gynecol Surv*. 2004;59(7):528-536. doi:10.1097/00006254-200407000-00022
95. Lachman E, Czernobilsky B, Ben-David M, Vlodavsky E, Lifschitz-Mercer B, Mammet Y. Prolonged retention of laminaria fragments: a rare complication of induced abortion. *Obstet Gynecol*. 2004;103(5 Pt 2):1128-1130. doi:10.1097/01.AOG.0000125150.64300.DD
96. Nilsson W, Mikhael S, Kaplan J. Chronic Pelvic Pain and Infertility Resulting from Unrecognized Retained Laminaria. *Case Rep Obstet Gynecol*. 2017;2017:1-3. doi:10.1155/2017/6345712
97. Bernstein SN, Cudemus-Deseda GA, Ortiz VE, Goodman A, Jassar AS. Case 33-2019: A 35-Year-Old Woman with Cardiopulmonary Arrest during Cesarean Section. *N Engl J Med*. 2019;381(17):1664-1673. doi:10.1056/NEJMCPC1904046
98. Benson MD. Amniotic fluid embolism mortality rate. *J Obstet Gynaecol Res*. 2017;43(11):1714-1718. doi:10.1111/jog.13445
99. Fong A, Chau CT, Pan D, Ogunyemi DA. Amniotic fluid embolism: antepartum, intrapartum and demographic factors. *J Matern Fetal Neonatal Med*. 2015;28(7):793-798. doi:10.3109/14767058.2014.932766
100. Kramer MS, Rouleau J, Baskett TF, Joseph K. Amniotic-fluid embolism and medical induction of labour: a retrospective, population-based cohort study. *Lancet (London, England)*. 2006;368(9545):1444-1448. doi:10.1016/S0140-6736(06)69607-4
101. Crissman HP, Loder C, Pancaro C, Bell J. Case report of amniotic fluid embolism coagulopathy following abortion; Use of viscoelastic point-of-care analysis. *BMC Pregnancy Childbirth*. 2020;20(1):1-7. doi:10.1186/S12884-019-2680-1/FIGURES/3
102. Panda S, Das A, Sharma N, Das R, Jante D vinayak. Amniotic Fluid Embolism After First-Trimester Abortion. *Cureus*. 2022;14(4). doi:10.7759/CUREUS.24490
103. Fekhkhar K, Rachet B, Gillet R, et al. Amniotic fluid embolism during curettage for a pregnancy arrest. Case report. *Ann Fr Anesth Reanim*. 2009;28(9):795-798. https://www.academia.edu/23616918/Embolie_amniotique_lors_d_un_curetage_pour_une_grossesse_arrêtée_À_propos_d_un_cas. Accessed June 28, 2022.
104. Rudra A, Chatterjee S, Sengupta S, Nandi B, Mitra J. Amniotic fluid embolism. *Indian J Crit Care Med*. 2009;13(3):129. doi:10.4103/0972-5229.58537
105. Ray BK, Vallejo MC, Creinin MD, et al. Amniotic fluid embolism with second trimester pregnancy termination: a case report. *Can J Anesth 2004 512*. 2004;51(2):139-144. doi:10.1007/BF03018773
106. Creanga AA, Syverson C, Seed K, Callaghan WM. Pregnancy-Related Mortality in the United States, 2011-2013. *Obstet Gynecol*. 2017;130(2):366-373. doi:10.1097/AOG.0000000000002114
107. Zane S, Creanga AA, Berg CJ, et al. Abortion-Related Mortality in the United States: 1998-2010. *Obstet Gynecol*. 2015;126(2):258-265. doi:10.1097/AOG.0000000000000945
108. Clark SL, Romero R, Dildy GA, et al. Proposed diagnostic criteria for the case definition of amniotic fluid embolism in research studies. *Am J Obstet Gynecol*. 2016;215(4):408-412. doi:10.1016/J.AJOG.2016.06.037
109. Moore J, Baldisseri MR. Amniotic fluid embolism. *Crit Care Med*. 2005;33(10 Suppl). doi:10.1097/01.CCM.0000183158.71311.28
110. Kaur K, Bhardwaj M, Kumar P, Singhal S, Singh T, Hooda S. Amniotic fluid embolism. *J Anaesthesiol Clin Pharmacol*. 2016;32(2):153-159. doi:10.4103/0970-9185.173356
111. Creel-Bulos C, Hassani B, Stentz MJ, et al. Extracorporeal Membrane Oxygenation for Amniotic Fluid Embolism-Induced Cardiac Arrest in the First Trimester of Pregnancy: A Case Report. *Crit care Explor*. 2020;2(7):e0162. doi:10.1097/CCE.0000000000000162
112. Adachi M, Adachi T, Fujita T, Hyuga S, Onishi Y, Okutomi T. Venoarterial extracorporeal membrane oxygenation as an early treatment for amniotic fluid embolism with cardiac arrest: A case report. *J Obstet Gynaecol Res*. 2021;47(9):3374-3378. doi:10.1111/JOG.14880
113. Aissi James S, Klein T, Lebreton G, et al. Amniotic fluid embolism rescued by venoarterial extracorporeal membrane oxygenation. *Crit Care*. 2022;26(1). doi:10.1186/s13054-022-03969-3
114. Austin J, Ford MD, Rouse A, Hanna E. Acute intravaginal misoprostol toxicity with fetal demise. *J Emerg Med*. 1997;15(1):61-64. doi:10.1016/S0736-4679(96)00257-0
115. Bentov Y, Sheiner E, Katz M. Misoprostol overdose during the first trimester of pregnancy. *Eur J Obstet Gynecol Reprod Biol*. 2004;115(1):108-109. doi:10.1016/J.EJOGRB.2003.10.004
116. Barros JG, Reis I, Graça LM. Acute misoprostol toxicity during the first trimester of pregnancy. *Int J Gynaecol Obstet*. 2011;113(2):157-158. doi:10.1016/J.IJGO.2010.12.006
117. Randall Bond G, Van Zee A. Overdosage of misoprostol in pregnancy. *Am J Obstet Gynecol*. 1994;171(2):561-562. doi:10.1016/0002-9378(94)90302-6
118. Graber DJ, Meier KH. Acute misoprostol toxicity. *Ann Emerg Med*. 1991;20(5):549-551. doi:10.1016/S0196-0644(05)81614-2
119. Gottlieb M, Long B, Koyfman A. The Evaluation and Management of Toxic Shock Syndrome in the Emergency Department: A Review of the Literature. *J Emerg Med*. 2018;54(6):807-814. doi:10.1016/J.JEMERMED.2017.12.048
120. Nikolic O, Nikolic MB, Dar Spasic A, Otero-Garcia MM, Stojanovic S. Systematic radiological approach to uteroovarian pathologies. *Br J Radiol*. 2019;92(1099). doi:10.1259/BJR.20180439/SUPPL_FILE/BJR.20180439.SUPPL-01.DOCX
121. Scarvelis D, Malcolm I. Embolization of a huge tricuspid valve bacterial vegetation. *J Am Soc Echocardiogr*. 2002;15(2):185-187. doi:10.1067/MJE.2002.118173
122. Andresen K, Balf D, Marcu CB. Images in cardiology: Massive pulmonary embolism in a patient with Streptococcus agalactiae tricuspid valve endocarditis. *Heart*. 2005;91(3):279. doi:10.1136/hrt.2004.038828
123. Reardon DC. The abortion and mental health controversy: A comprehensive literature review of common ground agreements, disagreements, actionable recommendations, and research opportunities. *SAGE open Med*. 2018;6:205031211880762. doi:10.1177/2050312118807624
